# Mapping the ethical and sustainable transition in toxicology: a bibliometric analysis and a review of new approach methodologies

**DOI:** 10.1007/s00204-025-04209-7

**Published:** 2025-10-02

**Authors:** Ruxandra Malina Petrescu-Mag, Mathieu Vinken, Dacinia Crina Petrescu

**Affiliations:** 1 Faculty of Environmental Science and Engineering, Babes-Bolyai University, 30 Fantanele Street, 400294 Cluj-Napoca, Romania; 2 Doctoral School “International Relations and Security Studies”, Babes-Bolyai University, 1 M. Kogalniceanu Street, 400084 Cluj-Napoca, Romania; 3 Department of Economy and Rural Development, Faculty of Gembloux Agro-Bio Tech, University of Liège, Passage Des Déportés 2, 5030 Gembloux, Belgium; 4 Department of Pharmaceutical and Pharmacological Sciences, Vrije Universiteit Brussel, Laarbeeklaan 103, 1090 Brussels, Belgium; 5 Faculty of Business, Babes-Bolyai University, 7 Horea Street, 400174 Cluj-Napoca, Romania

**Keywords:** 3Rs principle, Animal welfare, In silico models, In vitro toxicology, NAMs, Public perception

## Abstract

Toxicology is undergoing a paradigm shift, driven by the ethical imperative to reduce animal testing, the pursuit of sustainability, and regulatory transitions toward new approach methodologies (NAMs). This study systematically maps the integration of ethics and sustainability into NAMs-related toxicological research, using a mixed-methods design that combines bibliometric analysis with a review of scientific and policy literature. Our findings reveal a steep increase in NAMs publications since 2015, with in vitro and in silico approaches at the forefront. Bibliometric clustering identified three dominant thematic domains—regulatory testing, methodological performance factors, and human cell culture innovation—each reflecting varying degrees of engagement with ethical, scientific, and sustainability principles. A qualitative matrix was also developed to link the bibliometric clusters to key ethical and methodological dimensions, highlighting the growing centrality of themes such as the 3Rs, sustainability, and regulatory reform. Notably, the scientific and political discourse is shifting from merely “symbolic” ethics, used primarily to signal alignment with funding priorities or public expectations, toward more deeply embedded and actionable ethical frameworks. Initiatives emphasize operational ethics through concepts such as the fourth R (responsibility), with more expanded models including 12Rs, the 3C model (cell culture, computer simulation, and clinical trials), and ethics-driven AI tools. These developments signal a maturing field where ethics is becoming a methodological imperative. By mapping these shifts, the study offers an integrated perspective on how ethical values shape scientific innovation in toxicology. It provides evidence-based directions for accelerating a responsible transition to animal-free, human-relevant, and resource-efficient risk assessment.

## Introduction

In recent decades, toxicology has experienced a transformation toward more ethical, efficient, and human-relevant methodologies. At the forefront of this transition are new approach methodologies (NAMs), an umbrella term encompassing non-animal approaches, including in vitro (cell- or tissue-based laboratory experiments), in silico (computational or AI-driven modeling), *in chemico* (chemical reactivity-based assays), and integrated strategies that, individually or in combination, enhance chemical safety assessment by providing more relevant or protective models, thereby supporting the reduction and eventual replacement of animal testing (Colbourne et al. 2025; Schmeisser et al. 2023; Sewell et al. 2024; Vinken 2024). As defined by the US EPA, NAMs include “any technology, methodology, approach (including computational/in silico models like QSARs—quantitative structure–activity relationship), or combination thereof that can be used to provide information on chemical hazard and risk assessment that avoids the use of intact animals” (EPA 2024). Regulatory and scientific institutions, including the OECD or the European Medicines Agency adopted this definition (European Medicines Agency 2025a; OECD 2020). NAMs may include previously established scientific techniques; however, what distinguishes them as “new” is their targeted application within regulatory frameworks and their intentional design to replace, reduce, or refine the use of animal testing (Basu et al. 2025). Furthermore, NAMs are driving innovation by advancing scientific and technological frontiers, thereby accelerating and deepening our understanding of how toxicants affect both human health and environmental systems (Basu et al. 2025).

NAMs are inherently interdisciplinary, comprising advanced cell-based assays, organoids, organ-on-chip systems, omics technologies, QSAR modeling, and integrated approaches to testing and assessment (IATA), the latter being formal decision frameworks that combine diverse data types to draw regulatory conclusions. IATA are particularly relevant in regulatory toxicology, as they offer structured, weight-of-evidence approaches for chemical safety assessments (OECD 2020).

**Fig. 1:**
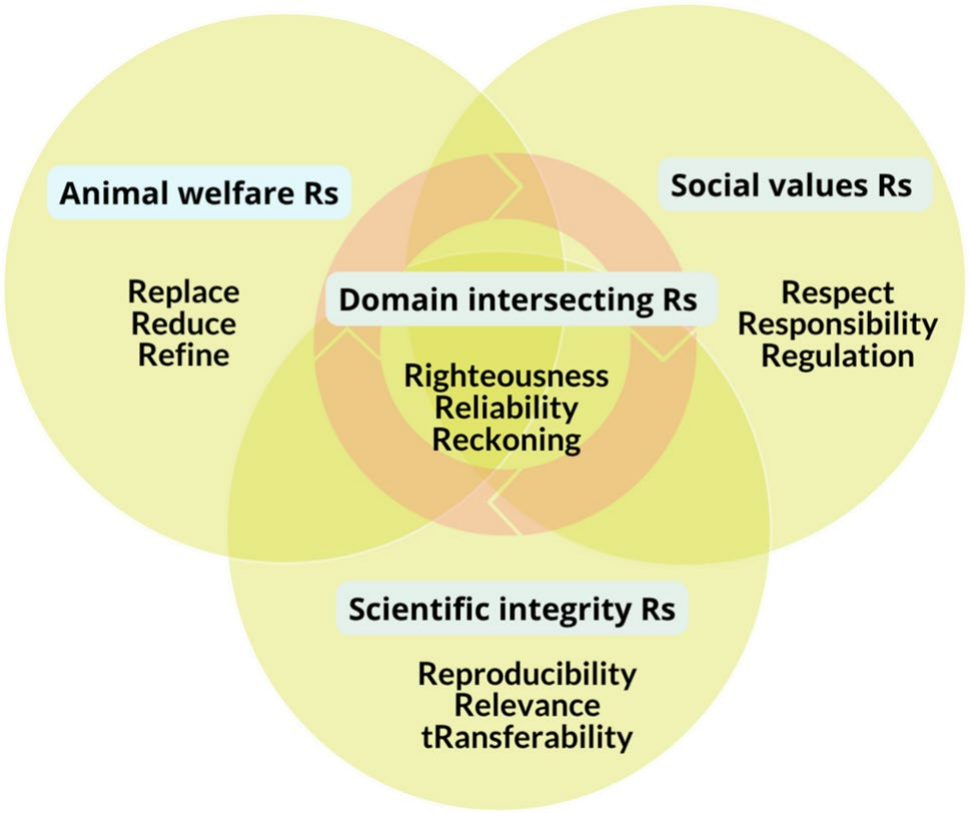
A simplified 12 Rs framework for the ethical treatment of animals in scientific research. Source: adapted after Brink and Lewis(2023)

The ethical motivation for NAMs originates from the 3Rs principle—replacement, reduction, and refinement—formulated by Russell and Burch (1959) and institutionalized in European legislation via Directive 2010/63/EU (The European Parliament and the Council 2010). This principle mandates that animal use in scientific research be minimized or replaced with alternatives whenever scientifically feasible (Grimm et al. 2023). However, the continued use of animals in research, despite scientific progress, has raised ethical concerns about whether the 3Rs are enough. Critics argued that institutional inertia, regulatory conservatism, and lack of accountability hinder the actualization of the 3Rs (Petetta and Ciccocioppo 2021). To address these limitations, the ethical framework has been extended to include a fourth R, namely, responsibility, which emphasizes moral agency, proactive engagement, and a culture of care in research settings (Grimm et al. 2023; Leist et al. 2012; Liu et al. 2025), also referred to sometimes as “Refusal” (of unethical or scientifically unjustified protocols). Some scholars proposed ever larger frameworks, with 12Rs and spanning several ethical domains (animal welfare, social value, scientific integrity, and domain-intersecting Rs (Brink and Lewis 2023) (see Fig. 1).

Animal welfare remains central to the NAMs discourse. Public resistance to animal testing, fueled by heightened awareness and activism, led to political momentum in the USA and the EU to reform legislation and accelerate the transition to alternatives. The recently published Horizon Scanning Report on NAMs (European Medicines Agency 2025b) emphasizes that achieving these goals requires a more substantial alignment of innovation policies, risk governance frameworks, and long-term investment strategies.

In parallel, sustainability has become a driving force behind the adoption of NAMs. Traditional toxicity testing relies heavily on live animals, is resource-intensive, and produces considerable biological and chemical waste. By contrast, NAMs offer low-waste, high-throughput, and energy-efficient alternatives that align with the EU’s Green Deal and Zero Pollution Action Plan. Furthermore, NAMs support the circular economy and green toxicology by promoting safer chemical design and earlier hazard identification using fewer resources (Crawford et al. 2017; Zorzan et al. 2022).

Despite their promise, the broad implementation of NAMs remains challenged by validation hurdles, regulatory conservatism, and a lack of harmonized guidance (Serafini et al. 2024). Coordination and engagement of multiple stakeholders are required to accelerate the acceptance and effective use of NAMs (Hoogstraaten et al. 2025). For example, the OECD (OECD 2005, 2020) reports highlight the need for enhanced guidance on uncertainty assessment, integration methods, and mutual acceptance of data within IATA frameworks. The fragmented landscape of guidance documents and varying interpretations of core terms, such as “NAM”, “defined approach” (DA), and “weight of evidence”, continue to obstruct regulatory convergence (OECD 2020).

This study responds to a critical gap in the literature by examining how ethical considerations, particularly the imperative to reduce, refine, and ultimately replace animal testing, and sustainability principles, emphasizing resource efficiency, long-term societal impact, and responsible innovation, are reflected in the evolving scientific discourse on NAMs in toxicology. These dimensions are no longer peripheral, but are increasingly central to research priorities, regulatory strategies, and public expectations (Hartung 2024; Hartung and Tsaioun 2024).

To explore this shift, the study used a mixed-methods design combining bibliometric analysis (via VOSviewer) with a rapid review of the scientific literature to address the central research question (RQ): “How are ethical considerations and sustainability principles represented and integrated in the evolving scientific discourse on NAMs in toxicology?” In pursuit of this aim, the study sets out the following specific objectives:

(i) To systematically map and interpret the ethical and sustainability dimensions of NAMs-related toxicological research through advanced bibliometric techniques.

(ii) To analyze emerging trends, conceptual developments, and institutional positions within the scientific and strategic policy literature.

(iii) To generate evidence-informed insights that can support a responsible, science-based transition toward animal-free and sustainable toxicological practices.

The present study offers a novel contribution by bridging bibliometric mapping with ethical and sustainability analysis, an interdisciplinary perspective often missing in NAMs-related reviews. As Hartung (2024) argues, rethinking validation through ethical and philosophical lenses, by integrating principles such as beneficence, non-maleficence, and justice, can accelerate the responsible adoption of NAMs, aligning scientific progress with societal values and public trust. Moreover, the study provides a structured synthesis of how societal values and policy drivers are shaping the scientific development of toxicology, thus informing both future research agendas and regulatory strategies.

## Methodology

### Database selection

To map the ethical and sustainability dimensions of NAMs in toxicology, this study used a mixed-methods approach that combined bibliometric analysis and a review, applied to the same set of publications. Data was extracted from two multidisciplinary databases: Web of Science (WoS) and Scopus. While PubMed is central to biomedical literature, it does not provide comprehensive coverage of social sciences, ethics, or policy-related studies (AlRyalat et al. 2019). Considering the cross-sectoral scope of our study, which focuses on the ethical and sustainability aspects of NAMs rather than their mechanistic or biomedical features, we did not entirely exclude PubMed. Instead, we chose not to include the smaller, non-MEDLINE-indexed part of PubMed, which tends to be less relevant to the present research focus. MEDLINE, which forms the core of PubMed with its thoroughly curated biomedical content, is already covered through Scopus, our primary database, ensuring that we include sufficient relevant biomedical literature while allowing us to focus on broader ethical sources.

Furthermore, WoS and Scopus were selected due to their broader subject indexing (Martín-Martín et al. 2021) and their support for structured, reproducible, and multidimensional search strategies, which are vital for capturing interdisciplinary trends relevant to responsible innovation and animal-free testing. In addition, our analysis incorporates the “New Approach Methodologies EU-IN Horizon Scanning Report” published by the European Medicines Agency (2025a, b), which includes a bibliometric network analysis of NAMs-related publications in PubMed from 2009 to 2024, identifying a total of 42,616 documents. This further mitigates the need for direct inclusion of PubMed in our search strategy. Moreover, it is worth noting that Scopus already indexes the vast majority of PubMed content through its inclusion of MEDLINE.

### Search strategy and data processing

A single search and selection process (covering WoS and Scopus) was used to identify relevant literature. The resulting corpus was analyzed quantitatively (bibliometrically) using VOSviewer (Van Eck and Waltman 2023), to visualize key themes and relationships, and reviewed qualitatively (using the rapid review) to extract and synthesize insights on ethical and sustainability considerations in NAMs-related toxicology.

The literature search was performed in July 2025. Keywords were selected to capture specific technology terms (e.g., in vitro, computational), NAM terms, ethical framing, sustainability framing, and human applicability. For WoS, the following topic search (TS) (encompassing title, abstract, keywords) string was used, leveraging the platform’s support for phrase searching and wildcards: TS = (“new approach methodologies” OR NAM* OR “alternative testing” OR “non-animal testing” OR “animal-free” OR nonanimal OR replacement OR reduction OR refinement OR 3Rs) AND TS = (“in vitro” OR “cell culture” OR “Organ-on-Chip” OR computational OR “in silico”) AND TS = (ethic* OR “animal welfare” OR bioethics OR sustainability OR sustainable OR “green toxicology” OR “responsible innovation” OR “sustainability science”) AND TS = (human). This search yielded 971 documents without any filters applied for country or language. The predominant types of documents included articles (669) and reviews (244), with the top two subject categories being toxicology and environmental sciences.

For Scopus, we employed a simplified query to comply with platform limitations (maximum eight Boolean connectors per field, inconsistent handling of phrase searching and wildcards): (TITLE-ABS-KEY = (new approach methodologies OR 3Rs) AND TITLE-ABS-KEY = (in vitro OR in silico) AND TITLE-ABS-KEY = (ethics OR sustainability) AND TITLE-ABS-KEY = (human)). This returned 984 results. After excluding errata and a retracted article, the final dataset from Scopus consisted of 982 documents. The majority of these were also articles (212) and reviews (263), primarily from the fields of pharmacology, toxicology and pharmaceutics, and environmental science.

The publication time frame for the included literature spanned from 1998 to 2025 in Web of Science and from 2001 to 2025 in Scopus, reflecting over two decades of evolving discourse on non-animal testing.

All WoS and Scopus records were exported in RIS format and imported into Zotero, where 12 strategic documents from regulatory and institutional sources [e.g., (European Medicines Agency 2023, 2025b)] were also added. After deduplication, the merged dataset (initially comprising about 1965 records) was exported to Rayyan, a collaborative screening platform widely used for systematic reviews. Due to the high volume of retrieved documents and the substantial number of false-positive records (Petrescu-Mag et al. 2025), the papers that superficially matched search terms but lacked thematic relevance (e.g., studies on agriculture or unrelated biomedical assays) were excluded. We employed the Rayyan AI-assisted screening platform to streamline the exclusion of non-pertinent documents based on predefined inclusion criteria, a method increasingly recognized for improving transparency and efficiency in literature reviews (Ouzzani et al. 2016). In Rayyan, we reapplied the inclusion criteria based on thematic relevance to the following topics, e.g., NAMs, 3Rs principle, in vitro, in silico, alternative non-animal methods, ethics or animal welfare, responsible innovation, and sustainability. Records lacking sufficient relevance were excluded using a combination of keyword, title, and abstract screening. This process yielded a refined dataset of 333 publications, which was subsequently used for bibliometric analysis.

### Bibliometric analysis

To map the research landscape and identify conceptual linkages, we conducted a bibliometric analysis using VOSviewer (version 1.6.20) (Van Eck and Waltman 2023), a widely used tool for constructing and visualizing bibliometric networks.

The keyword co-occurrence map was generated using the binary counting method. Binary counting considers only the presence or absence of an item within each document. For example, if a keyword appears, it is counted as 1, and if not, it is counted as 0, regardless of how many times the keyword occurs. This method ignores frequency within the same document, making it especially suitable for keyword co-occurrence analysis, where repeated mentions of a term could otherwise distort the results. Binary counting is also ideal for thematic mapping, particularly when documents vary in length or focus. It ensures that each document contributes equally to the analysis, helping to avoid inflation caused by verbose or repetitive texts.

To ensure clarity and thematic relevance, we set a minimum keyword occurrence threshold of 7, striking a balance between comprehensiveness and specificity. This threshold aligns with bibliometric best practices, facilitating the exclusion of marginal or noise-generating terms while ensuring that potentially emerging topics are not prematurely excluded. Of the 11,345 terms identified across the dataset, 289 met the threshold. A relevance score calculated by VOSviewer then ranked these 289 terms. Following the default methodological recommendations (Van Eck and Waltman 2023), we selected the top 60% most relevant keywords to ensure a robust yet interpretable conceptual mapping, resulting in 173 terms.

The refined dataset was visualized using three types of VOSviewer maps (Van Eck and Waltman 2023). (a) Network visualization map: This map illustrates relationships among keywords through node-link structures. Node size reflects the frequency of a keyword, while the thickness of the link denotes the strength of co-occurrence. (b) Overlay visualization map: This map visualizes temporal trends in keyword usage, allowing the detection of emerging research directions. (c) Density visualization map: This map highlights thematic concentrations. Dense clusters represented mature research areas, while sparse regions suggested underexplored niches. Together, these visualizations enabled a systematic identification of research hotspots, interdisciplinary intersections, and potential gaps within the scholarly discourse on non-animal toxicology methods.

### The “rapid” review

Reviews serve to systematically gather, assess, and synthesize existing evidence to address a well‑defined research question (Uman 2011). One streamlined approach is the so-called “rapid” review (Hamel et al. 2021), also referred to as a restricted review in the taxonomy of Plüddemann et al. (2018). A rapid review aims to deliver timely and relevant synthesized evidence by accelerating the typical systematic review process through method simplification or omission, thus balancing speed and methodological rigor (Speckemeier et al. 2022). Rapid reviews emerged in response to the often prolonged and labor-intensive nature of systematic reviews, offering a practical solution (Speckemeier et al. 2022) and have often been used in various fields (Abraham et al. 2023; Meis-Harris et al. 2021; Petrescu-Mag et al. 2024; Stevens et al. 2025). When conducted appropriately, rapid reviews are recognized as credible and useful knowledge products (Plüddemann et al. 2018). In this study, a rapid review methodology was chosen as a pragmatic approach to generate robust evidence within limited time and resource constraints (Moons et al. 2021). The process followed the Cochrane Rapid Reviews Methods Group recommendations, including minimum methodological standards outlined by Garritty et al. (2021), as translated into Moons et al. (2021) guidance (see Table 1).

**Table 1:** Steps of the rapid review process used in this study

Step	Description
Setting the research question—topic refinement	The research question was defined as: “How are ethical considerations and sustainability principles represented and integrated in the evolving scientific discourse on NAMs in toxicology?” (As described in “Introduction”)
Setting eligibility criteria	Eligibility criteria are detailed in the “2.2 Search strategy and data processing” subsection of “Methodology”
Searching	The literature search was conducted in July 2025 by two independent reviewers
Documents selection	The study selection process is described in the “2.2 Search strategy and data processing” subsection of “Methodology” Title and abstract screening: • Two reviewers independently screened titles and abstracts • Discrepancies were resolved through discussion and consensus • Initial screening was facilitated by AI-assisted filtering using the Rayyan platform to exclude non-relevant documents based on predefined inclusion criteria • The final dataset included 333 publications, which were subsequently used for bibliometric analysis. These were screened for relevance (considering the title and abstract), and 49 documents were retained for rapid review
Data extraction for the analysis	The analysis considered a minimal, but relevant set of thematic categories (ethical considerations, human relevance, sustainability framing, and references to NAMs). The authors also identified the documents that corresponded to each cluster (analyzed in “4. Discussion”)
Risk of bias (RoB) assessment	The RoB in the included systematic reviews was evaluated by one researcher and independently checked by a second, using the ROBIS tool (Risk of Bias in Systematic Reviews), as described by Whiting et al. (2016). Any discrepancies in judgments were addressed through discussion until consensus was achieved. In line with the ROBIS guidance, the assessment focused on four key domains, each comprising five or six signaling questions. These domains were: (1) study eligibility criteria, (2) identification and selection of studies, (3) data collection and study appraisal, and (4) synthesis and findings. Response options for each item included “yes,” “probably yes,” “probably no”, “no”, and “no information”, with a “yes” response indicating low concern regarding potential bias
Synthesis	Evidence was synthesized narratively, in tables, and figures All authors contributed to the process

Table 2 in “Discussion” presents a qualitative synthesis based on interpretive reasoning, developed in response to the bibliometric clustering and thematic insights identified in the literature. Drawing on the three clusters produced through VOSviewer’s keyword co-occurrence analysis (“Testing”, “Factor”, and “Culture”), we analyzed how each cluster engages with key cross-cutting themes, including animal ethics, human relevance, scientific validity, regulatory integration, and sustainability. The level of emphasis assigned to each theme (e.g., “Core”, “Strong”, “Moderate”) reflects a form of abductive reasoning, an inferential approach commonly used in qualitative research to generate explanations by iteratively interpreting data patterns within and across conceptual domains (Elo and Kyngäs 2008; Krippendorff 2018). This approach enabled us to relate bibliometric structures to broader discursive and methodological trends, offering a structured yet flexible framework for cross-cluster interpretation**.**

**Table 2:**
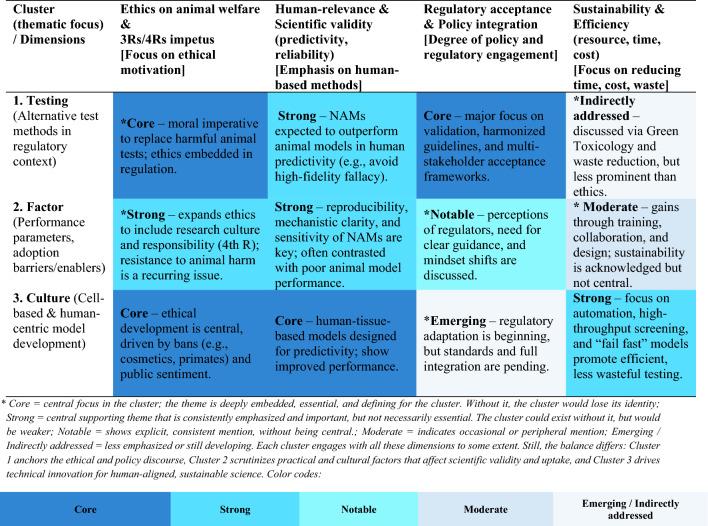
Qualitative matrix linking the bibliometric clusters to core ethical and methodological dimensions in the NAMs literature

## Results

### Co-occurrence network (network visualization)

The keyword co-occurrence network was visualized using VOSviewer, which grouped the 172 terms that met the minimum threshold of seven occurrences into three color-coded clusters, each representing a distinct thematic domain in the research on NAMs. The clustering was based on the frequency of co-occurrence and the semantic proximity of terms across documents. Each cluster is represented by a different color in the network visualization map, with the size of the nodes reflecting term frequency, and the number of links and total link strength indicating the degree of association that the most central (i.e., most connected) term has with other terms in the dataset. Specifically, “links” denote the number of distinct connections a term has with others. In contrast, “total link strength” refers to the cumulative weight of these connections, offering insight into how prominently the term figures in the network structure.

Cluster 1 (which we named “Testing”) has 86 terms, 154 links, and a total link strength of 734. This red-colored cluster was named for its clear concentration around terminology related to experimental assays, test system design, and regulatory pathways. The most central term in this cluster, “testing”, serves as a hub for related concepts such as “toxicity”, “cytotoxicity”, “regulatory acceptance”, “3R alternative method”, and “animal welfare.” These associations suggest that the cluster primarily reflects the methodological and compliance-related backbone of NAMs literature.

Cluster 2 (“Factor”) contains 49 terms, with 140 links and a total link strength of 411. Visualized in green, this cluster centers around the term “factor”, which is commonly associated with parameters that modulate outcomes in toxicity testing or experimental reproducibility. The cluster includes terms like “implication”, “optimization”, “efficiency”, “expression”, and “hazard.” The grouping reflects literature focused on performance parameters, interpretation contexts, and influencing variables in experimental and computational toxicology models.

Cluster 3 (“Culture”) comprises 37 terms, with 143 links and a total link strength of 587, and is shown in blue. We labeled this cluster based on the prominence of the concepts “culture,” “cell,” and “in vitro” as interconnected terms. Terms such as “stem cell,” “cell line,” “human cell,” and “tissue engineering” indicate a strong thematic orientation around cell-based methodologies and biological system modeling, highlighting the foundational methods used in NAMs to replicate or simulate human-relevant biological responses.

**Fig. 2:**
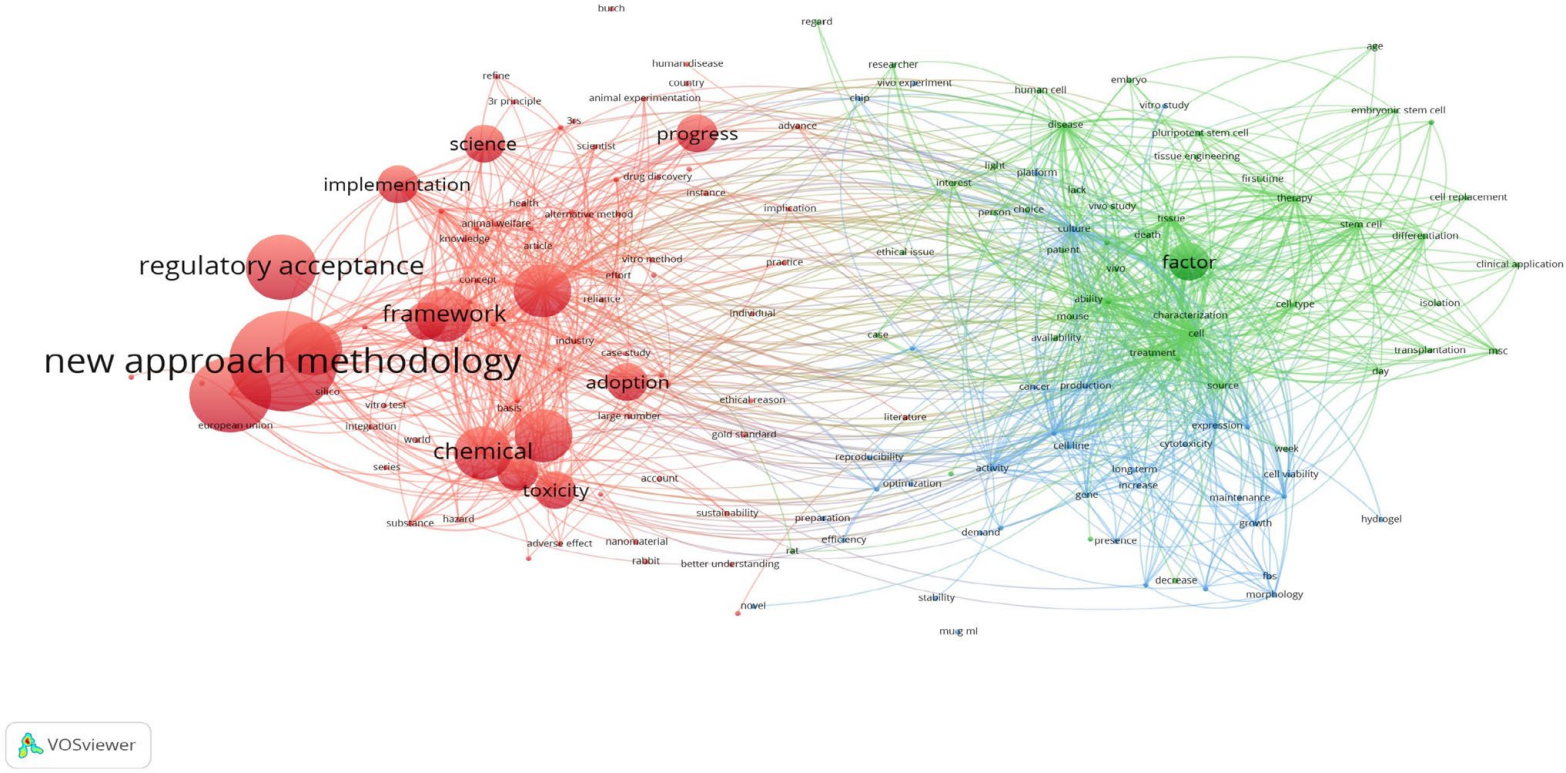
Network visualization map created in VOSviewer

Practically, the structure of the clusters reveals the conceptual segmentation of the field: the “Testing” cluster reflects standardized tools and regulatory context; “Factor” highlights variable-related reasoning and model behavior; and “Culture” captures the biological and technological substrates that underpin experimental platforms (Fig. 2).

### Temporal trends (overlay visualization)

**Fig. 3:**
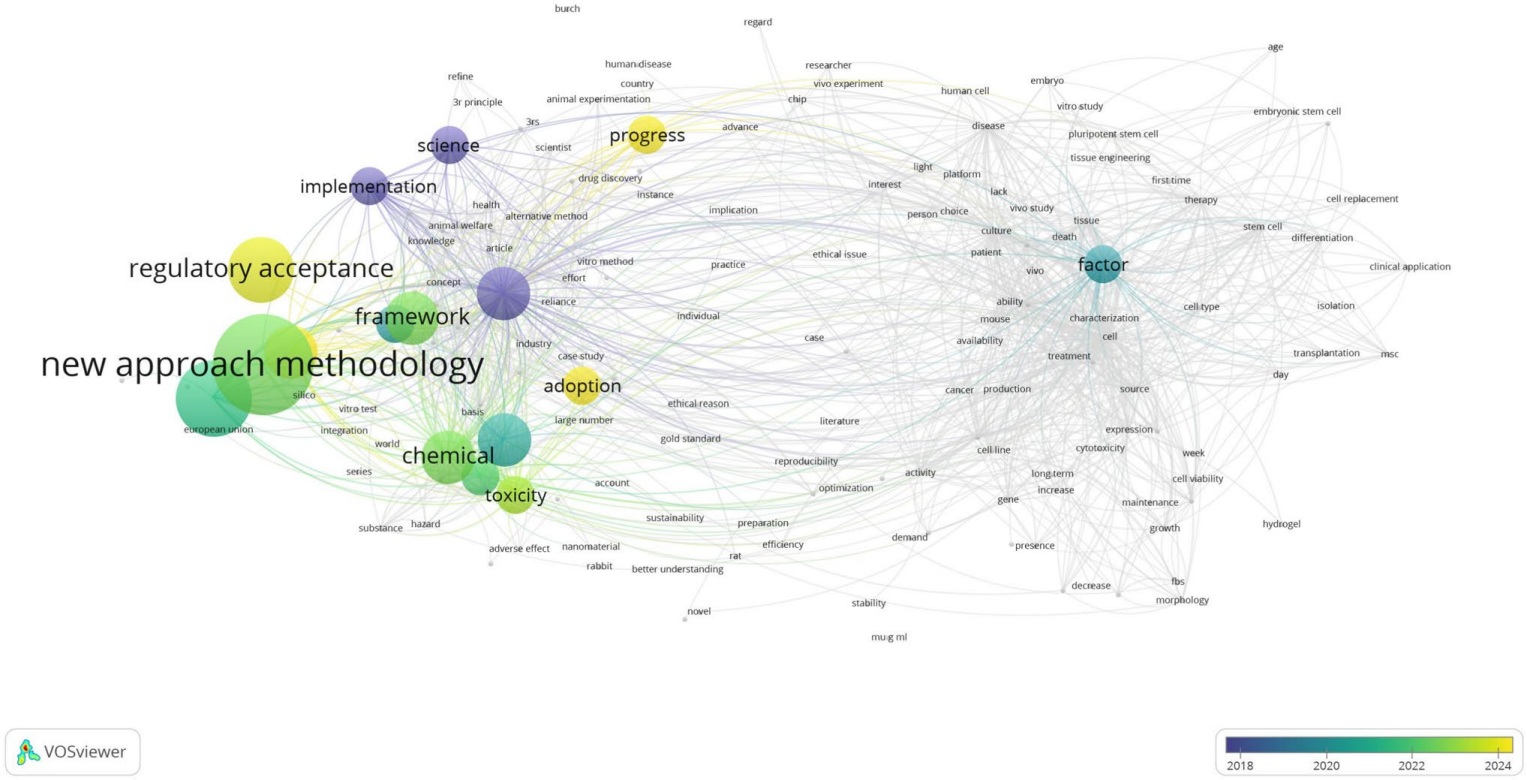
Overall visualization map created in VOSviewer

The overlay visualization map (Fig. 3) showed a temporal distribution of research activity. Earlier work (yellow to light green tones) centered around terms such as “animal experimentation”, “toxicity”, and “regulatory acceptance.” More recent terms (darker green to blue) included “responsible innovation,” “chip”, “nanomaterial”, and “ethical reason”, indicating a gradual shift toward socio-technical and integrative approaches in NAMs. Terms like “integration,” “sustainability,” and “framework” appeared in the later spectrum of the timeline, suggesting emerging directions that bridge methodological developments with broader ethical and policy dimensions.

### Thematic density (density visualization)

**Fig. 4:**
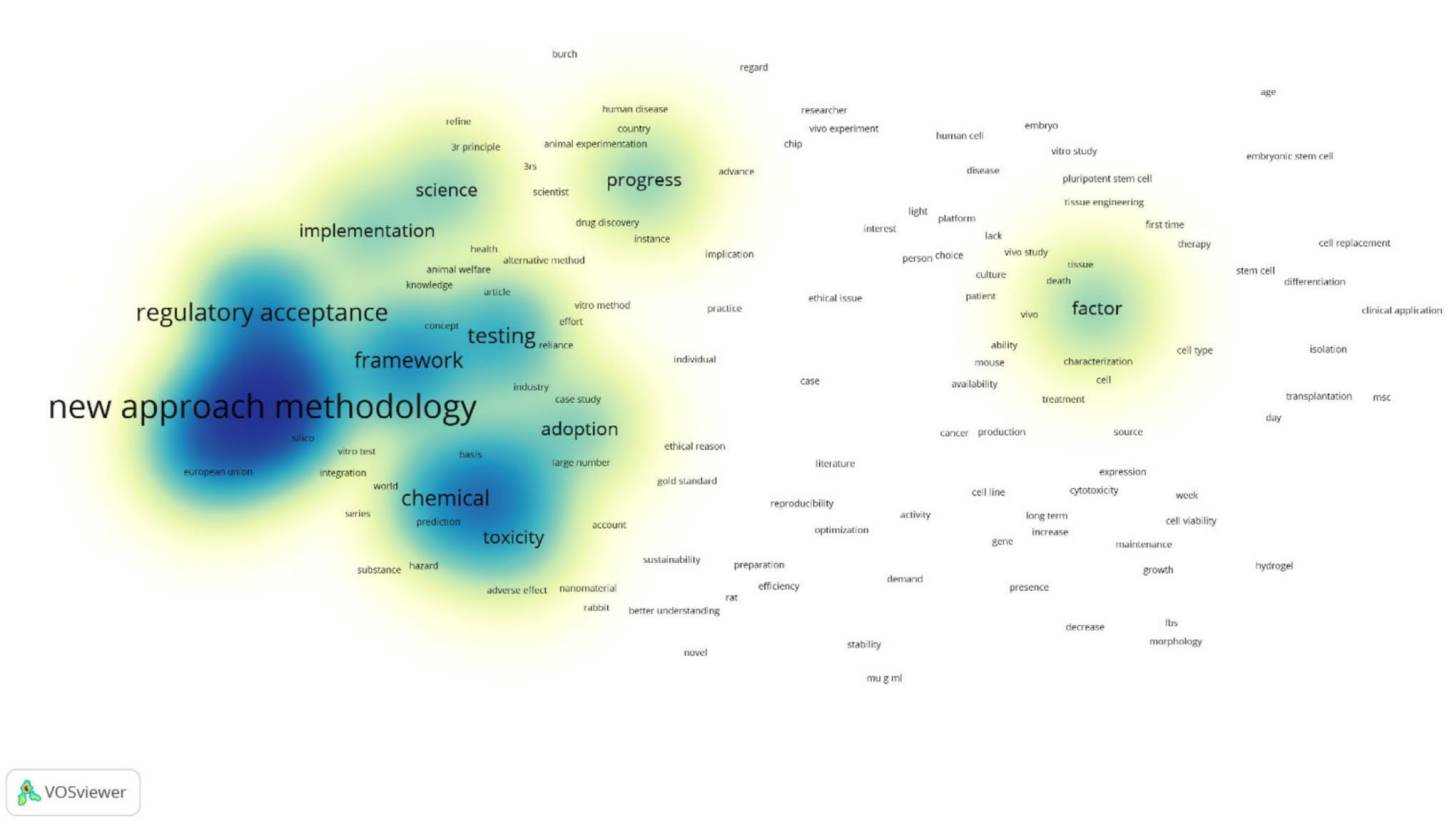
Density visualization map created in VOSviewer

The density visualization map (Fig. 4) revealed the concentration and intensity of research themes. Densely populated regions corresponded to well-established areas such as “in vitro”, “toxicity”, “animal welfare”, and “3Rs.” These were surrounded by moderately dense zones incorporating terms like “stem cell”, “drug discovery”, and “sustainability.” Peripheral, less dense clusters included emerging or specialized concepts such as “responsible innovation”, “chip”, “ethical issue”, and “green toxicology.” These areas suggest evolving but less mature lines of inquiry within the current research landscape.

## Discussion

### Cluster 1. “Testing”: integrating ethical drivers with regulatory innovation

The “Testing” cluster forms the core of the NAMs discourse, focusing on how new non-animal assays are accepted in regulatory toxicology. A central theme is the ethical obligation to replace animal tests (the 3Rs or 4Rs, and even the 12Rs) along with the practical need for reliable, human-relevant methods. Addressing these challenges is essential to unlock the full ethical and scientific potential of non-animal testing and to ensure fair, objective validation standards for alternative assays (Balls et al. 1990). Early advocates of replacement warned that institutional barriers and validation standards must be addressed to realize the ethical and scientific advantages of non-animal tests. Indeed, Balls (1994) concluded that greater effort was needed to overcome skepticism and ensure fair, objective validation of alternative assays, noting the “high fidelity fallacy”, the misplaced trust in animal models’ relevance to human risk. This ethical push for change, grounded in concerns about animal welfare and human translatability, laid the foundation for modern regulatory improvements. Today, international policy explicitly reflects these values. For example, the European Union’s 2010 Directive on animal use (The European Parliament and the Council 2010) embeds the 3Rs into law, and agencies such as the European Medicines Agency (EMA) (European Medicines Agency 2025c) and the U.S. FDA updated guidelines to promote 3R-aligned methods. In 2025, the FDA went far toward using more effective, human-relevant alternatives to replace animal testing in the development of monoclonal antibody treatments and other pharmaceuticals (FDA 2025). This regulatory evolution aligns with ethical obligations to minimize animal use and has been accelerated by public pressure and scientific evidence demonstrating that humane methods can enhance predictive accuracy.

A recurring insight in the “Testing” cluster is that ethics and scientific innovation are mutually reinforcing drivers of change. The authors describe a global convergence toward “animal-free” testing paradigms, motivated by both moral responsibility and the recognition of animals’ limited predictive power for human outcomes. For instance, Bos et al. (2020) argue that concerns about animal welfare and the poor human relevance of many animal models are driving forces behind new approaches in chemical safety assessment. Similarly, Poh and Stanslas (2024) recount how the 3Rs principle has stimulated policy changes worldwide, leading to more human-relevant in vitro technologies that improve welfare without sacrificing scientific rigor. Across regulatory domains (from pharmaceuticals and pesticides to chemicals and cosmetics), there is an ongoing shift toward integrating NAMs into formal safety evaluation frameworks, supported by guidance documents and strategic roadmaps that encourage their use (Bearth et al. 2025). Bearth et al. (2025), for example, surveyed European risk assessors and found growing familiarity with specific NAMs tools (e.g., QSAR models), but also highlighted heterogeneity in usage and confidence across sectors. They identified barriers and enablers for the acceptance of NAMs, notably the need for more explicit guidance and success stories to build trust in new methods. In line with this, recent reviews revealed that regulatory agencies are adopting more flexible, fit-for-purpose validation criteria to evaluate NAMs (Clippinger et al. 2018; Hartung 2024), as long as the methods demonstrate reliability and “human-relevant” predictive value equal to or greater than that of traditional animal tests. Stucki et al. (2022) report that regulatory frameworks in the USA, Canada, and the EU now explicitly permit NAMs data, and collaborative efforts (e.g., the OECD and the Interagency Coordinating Committee on the Validation of Alternative Methods workgroups) are focused on improving validation processes to accelerate the regulatory uptake of feasible alternatives. In short, this cluster reveals a broad consensus that ethical and scientific imperatives are aligning: regulators and scientists increasingly view NAMs as morally preferable and as an opportunity to improve the efficiency and human relevance of toxicity testing.

Notably, sustainability emerges as a complementary concern in the “Testing” cluster alongside ethics and innovation, making toxicology more sustainable in a broader sense and enhancing efficiency in chemical risk assessment (Hoffmann et al. 2016). Traditional toxicity testing is resource- and animal-intensive, and recent initiatives position “Green toxicology” as a strategy for safer chemical design that inherently reduces animal use and environmental waste. Maertens et al. (2014) consider that green toxicology requires, in addition to scientific advancements, a shift in corporate culture that fosters collaboration between synthetic chemists and toxicologists. Crawford et al. (2017) introduced green toxicology as a framework that promotes early-stage safety assessments with computational methods, thereby limiting the volume of hazardous chemicals synthesized and tested while also diminishing reliance on animal assays. This coupling of sustainable chemistry with NAMs-driven testing exemplifies how sustainability and ethics converge: reducing animal testing often coincides with reducing chemical waste and energy, making toxicology more sustainable in a broader sense.

At a policy level, the EU’s Chemicals Strategy for Sustainability (European Commission 2020) explicitly calls for innovating hazard assessment “to reduce dependency on animal testing” as part of achieving a toxic-free environment. Carnesecchi et al. (2023) emphasize that under the Strategy, regulators aim to enhance chemical safety and minimize unnecessary animal use by harmonizing data requirements. One example is the development of standardized OECD Harmonized Templates for NAMs data reporting, which facilitates cross-agency reuse of non-animal test results. Thus, the push for NAMs is framed as an ethical choice and as part of a sustainability transition in toxicology, aiming to improve efficiency and transparency in chemical risk assessment.

A key insight from this cluster is that realizing an “animal-free” future requires coordinated innovation in multiple areas. Scientifically, new methods must be optimized and validated to ensure they are truly suitable for their intended purposes. Institutionally, regulators and stakeholders need to coevolve practices and acceptance criteria. Several papers highlight the ongoing work to bridge these gaps. For example, a multi-stakeholder team led by Parish et al. (2020) developed a common evaluation framework for NAMs, as a structured set of criteria to assess whether a given NAM produces information adequate for regulatory decisions. The goal of such frameworks is to build confidence across industries and authorities, unifying disparate validation efforts and accelerating the incorporation of credible NAMs into safety assessments. Likewise, numerous international collaborations [e.g., EU-NETVAL, ICCVAM, etc., see Poh and Stanslas (2024)] are intensifying, aimed at standardizing new assays and achieving global regulatory acceptance of NAMs as quickly as possible. These initiatives build on earlier proposals for modular, structured evaluation frameworks (Hartung et al. 2004). However, the authors warn that policy alone is not a panacea. Cultural inertia and knowledge gaps can slow adoption even where regulations permit NAMs data. The literature, therefore, calls for sustained “top-down and bottom-up” efforts: high-level mandates and funding (e.g., large EU programs like ONTOX for next-generation risk assessment, part of the ASPIS Cluster, see Vinken et al. 2021) combined with grassroots changes in scientific practice and education. Practically, Cluster 1 (“Testing”) depicts a field in transition, driven by ethical and sustainability mandates to replace animal tests, and enabled by regulatory innovation and collaborative frameworks that seek to normalize NAMs in safety science. The cluster’s dense network of terms like “regulatory acceptance”, “alternative method”, “toxicity”, and “animal welfare” reflects how tightly intertwined these themes are: achieving regulatory acceptance of alternative methods is both a scientific challenge and an ethical imperative. Each success in validating a non-animal test or updating a guideline not only contributes to better science, but also advances the goal of a more humane and sustainable toxicology.

Building on this, numerous studies further demonstrate how technological advances are translating ethical principles into actionable testing strategies. For example, organoid systems, organ-on-chip platforms, and transgenic models are increasingly enabling 3R-compliant safety testing across pharmaceuticals, vaccines, and endocrine disruptors (Isbrucker et al. 2011; Jin et al. 2020; Penza et al. 2009). The integration of advanced in vitro models and computational simulations has proven to reduce animal use and to increase predictive value, especially in neurovirulence, ocular, and skin toxicity testing (Robinson et al. 2002; Ubels and Clousing 2005). While some challenges remain regarding the complexity of human physiology or the scalability of advanced cell-based models (Manful et al. 2023; Mehmood et al. 2024), the consensus is clear: alternatives grounded in human-relevant biology (supported by EU-funded frameworks and legislation like Directive 2010/63/EU) are the most promising path forward (Grimm et al. 2023; Langbein et al. 2020). As Roy et al. (2025) argue, the strategic convergence of cell culture, computational tools, and early-phase clinical approaches (the “3C solution”) may render large-scale animal use unnecessary in the long term. The ONTOX project (Vinken et al. 2021) exemplifies this direction by combining AI-driven predictive modeling with mechanistic in vitro data to eliminate the need for systemic animal testing in chemical safety assessments. Together, these perspectives demonstrate that the ethical drivers discussed in Cluster 1 are shaping a new testing paradigm that is both humane and scientifically rigorous, while also being regulatory aligned.

### Cluster 2. “Factor”: addressing technical and cultural factors for NAMs adoption

The “Factor” cluster highlights the variables and contextual considerations that influence the performance, interpretation, and acceptance of NAMs. Unlike the other clusters, which focus on broad methodologies or specific laboratory techniques, this cluster focuses on the conditions that enable or hinder the transition to animal-free testing. Key terms such as “factor”, “optimization”, “efficiency”, “implication”, and “hazard” indicate a preoccupation with the parameters that influence outcomes, whether those outcomes are experimental results or regulatory decisions. In essence, Cluster 2 addresses the scientific factors (reproducibility, validity, sensitivity) and the human factors (perception, familiarity, stakeholder behavior) that must be managed to realize the full potential of NAMs.

A prominent theme in this cluster is improving the scientific robustness and relevance of new methods. Several sources emphasize that NAMs must not only exist, but also perform well and be reliable to gain trust. For example, Mondou et al. (2020) examined the ecotoxicology community and found that experts’ perception of a NAM’s viability strongly depended on their familiarity with it, a phenomenon termed the “pattern of familiarity”. Long-practicing toxicologists tended to trust conventional animal tests or NAMs with which they were familiar, while newer professionals were more open to innovative techniques. Moreover, respondents expressed concern about “error costs”, in the sense of the risk of wrong decisions if a new method fails, which made some regulators more skeptical of NAMs data. This insight underscores that, beyond developing new methods, the field must actively build expertise and confidence in its use. Education and training emerge as critical factors. Costin and Miller (2022) argue that introducing the 3Rs and NAMs concepts early in scientific education can produce a new generation of researchers who are familiar with modern toxicology tools. Their results from a survey of teachers and students revealed a strong interest in learning non-animal methods and the ethical context of testing, suggesting that curriculum reform is a viable way to shift the research culture toward the acceptance of NAMs. Similarly, Herrmann et al. (2019) call for educational efforts targeting young scientists to accelerate a future where high-tech replacements (e.g., organs-on-chips, computational models) become the default in biomedical research. Together, these perspectives highlight “familiarity” and training as key sociological factors in the adoption of technology. It can be inferred that scientists and regulators are more likely to trust and adopt NAMs when they understand them and have seen them work, which means investing in cross-sector knowledge exchange and capacity building is just as important as technical R&D.

On the technical side, Cluster 2 literature illustrates the optimization of NAMs performance and credibility. Ensuring that non-animal models are as predictive and reproducible as possible is a recurring concern. Emerson (2010) illustrates how applying the 3R principle in cardiovascular research led to the development of refined animal models that not only used fewer animals, but also produced better scientific outcomes. By reducing severity and improving experimental design (e.g., refining thrombosis models in mice), researchers obtained more reliable data and gained more profound insights into disease mechanisms. This example demonstrates that refining methodologies (a “factor” within experiments) can align ethical and scientific goals and yield higher-quality results while minimizing harm. In many cases, the challenge lies in replicating complex biology without using animals, and authors discuss creative strategies to achieve this. Jang et al. (2025) provide a compelling case study. They developed a hybrid QSAR model incorporating mechanistic toxicology data (like enzyme binding affinities from molecular docking) to predict lethal doses of nerve agents. Because ethical and safety barriers make it impossible to determine these lethal doses in animals or humans, a mechanism-driven in silico approach was crucial. The resulting model could predict the toxicity of untested compounds (including nerve agents) and thus stands as an ethically sound and scalable alternative to lethal animal tests, provided its predictions meet regulatory standards. The success of this approach relied on integrating key biological “factors” (enzyme interactions) into the computational model, thereby improving its relevance. Similarly, Staumont et al. (2025) introduce the concept of physiological maps (detailed, curated networks of biological processes) to bolster mechanistic understanding in NAMs. They argue that many current in silico models lack physiological grounding. By mapping gene and cell-level events (like an adverse outcome pathway framework), one can design in vitro and in silico tests that more accurately emulate human biology. This work exemplifies a technical optimization factor: mechanistic detail, which can enhance the credibility of NAMs in hazard assessment. In practice, such advances translate into more accurate predictions of human-relevant hazards, helping to convince stakeholders that NAMs are not “black boxes”, but scientifically robust tools.

Beyond the laboratory, cultural and organizational factors are emphasized in this cluster. Resistance to change, whether due to inertia, habits, or institutional norms, is identified as a non-technical hurdle to NAMs implementation. Sewell et al. (2024) explicitly discuss how “comfort with established methods” can hinder the shift to NAMs. Decades of historical animal data and expertise create a status quo bias. Many toxicologists feel confident about animal models due to their long-standing familiarity and the extensive baseline of reference data. To counter this, Sewell and colleagues suggest that NAMs proponents must demonstrate clear benefits (e.g., financial, productivity, or scientific) to persuade institutions to invest in change. Crucially, they note that a “mindset shift” is needed. Thus, regulators and scientists should focus on how NAMs-based decisions can be protective of human health rather than trying to mimic animal test results one to one. In other words, the community must accept that new methods may not directly replicate animal data, but can still be valid if they effectively predict human outcomes or safety thresholds. This cultural reframing, which views NAMs as different, but not deficient, is an important factor in promoting progress. It aligns with the arguments presented by Burgdorf et al. (2019), who posed the question of “evolution versus revolution” in validation approaches. They highlight that while an incremental, evolutionary validation of NAMs (comparing them to animal tests) was the norm, a more “revolutionary” mindset might be required, one that reconsiders what standards of evidence are truly needed for safety decisions, rather than holding NAMs to the legacy animal benchmark in every case.

Another cross-cutting dimension in Cluster 2 is stakeholder collaboration and responsibility. Because adopting NAMs often involves multiple sectors (academia, industry, regulators, NGOs), better collaboration is seen as a factor that can accelerate acceptance. Bos et al. (2020) argue for multi-stakeholder engagement early in method development so that regulatory needs (e.g., data requirements, validation criteria) shape the design of NAMs. This ensures new methods are “born” meeting the standards that will later be expected, smoothing the path to uptake. At the same time, the ethical dimension of responsibility is being expanded. Liu et al. (2025) propose a fourth R from “responsibility” to complement the traditional 3Rs in research contexts where animals are still used. This principle emphasizes the duty of individual researchers to proactively seek alternatives and minimize harm when animal use is unavoidable. By incorporating personal accountability (e.g., through ethics training, enhanced oversight, and a culture of care) into the framework, Liu et al. (2025) consider that the community can more effectively challenge outdated practices and promote the adoption of humane methods. The introduction of “Responsibility” as a cultural factor reflects a broader trend. It refers to the fact that scientists are not only developing NAMs, but also examining their practices and biases in adopting them. This introspective angle highlights the importance of ethical leadership within the scientific community, complementing policy mandates. Historical examples (like the development of life-saving therapies using animal models under dire circumstances) are acknowledged by Liu et al. (2025). Still, they use these cases to argue that moving forward, every effort must be made to balance scientific progress with empathy and respect for life.

In summary, Cluster 2 (“Factor”) emphasizes that the transition to NAMs does not occur automatically with the invention of new methods. Instead, it depends on the convergence of multiple enabling factors. Scientifically, methods must be optimized for relevance and reproducibility (addressing issues of mechanistic validity, sensitivity, and domain of applicability). Logistically, validation and data-sharing processes should be improved and possibly reimagined, so that promising NAMs can be evaluated efficiently without defaulting to animal comparisons in every case. Culturally, scientists, risk assessors, and institutions require greater exposure to NAMs successes (through training, pilot studies, and the communication of success stories) to build trust (Bearth et al. 2025). Social science research, such as that of Mondou et al. (2020), suggests that as familiarity increases and cross-sector collaboration grows, confidence in NAMs also increases. Encouragingly, we see this happening. Workshops and surveys report shifting attitudes, with many experts believing that NAMs are nearing viability for broad use. Nonetheless, the cluster’s focus on “factors” reminds us that barriers remain, whether they be technical limitations in current models or psychological and institutional reluctance. Overcoming these will require continued refinement of the methods (e.g., advancing organoid systems and improving computational model interpretability) and deliberate strategies to change the practice and mindset of toxicology. Ethics and sustainability continue to emerge as underlying motives. For example, optimizing tests to require fewer animals or less reagents not only improves efficiency, but also reduces ethical costs and environmental footprint (MeridianBioscience 2025). Thus, Cluster 2 draws attention to the nuanced, often-overlooked efforts across laboratories, boardrooms, and classrooms that collectively support the overarching ethical and sustainable transition explored in this review.

### Cluster 3. “Culture”: advancing cell-based models and human biomimicry

The “Culture” cluster centers on the laboratory heart of new approach methodologies: cell cultures, tissue engineering, organoids, microphysiological systems, and related in vitro innovations that aim to replicate human biology. With terms such as “culture”, “cell”, “in vitro”, “stem cell”, and “tissue engineering”, this cluster reflects the research into biological substrates that can model human-relevant responses without the use of live animals. These technologies are the practical means by which the ethical and conceptual goals discussed in the other clusters are realized. In the “Culture” cluster, ethics and sustainability manifest as design criteria: new models are valued for being human-relevant and humane, and their development is often justified by the ability to reduce animal use and accelerate discovery in a cost-effective way.

A central theme in this cluster is the remarkable progress in reproducing human physiology in vitro. Over the past decade, advanced systems such as 3D organoids, induced pluripotent stem cell (iPSC) derivatives, and organ-on-chip devices have moved from experimental curiosities to mainstream research tools. Lee et al. (2025) reviewed these developments, noting that stem cell-based models and organoids emerged as revolutionary alternatives that more accurately mimic human organ functions, from liver metabolism to brain microarchitecture. The advantage of these models lies in their dual promise: they are ethically advantageous (derived from consenting human cell sources or reprogrammed cells, thus avoiding animal sacrifice) and often scientifically superior, as they capture species-specific biology that animal models cannot. For example, human organoids can recapitulate complex features, such as organ development or disease pathology, in vitro, offering predictive insights into drug efficacy and toxicity that are directly relevant to patients. Multiple studies echo this point. Neziri et al. (2024) highlight that in neuroscience and other fields, animal models frequently fail to translate to human success—over 90% of drugs successful in animals do not work in humans (Garner 2014). Neziri et al. (2024) report that human-based models (e.g., neural organoids combined with computer modeling) are being pursued to fill this gap, not only to meet ethical expectations but also to improve predictive accuracy in complex diseases. Likewise, Costa et al. (2021) and Luconi et al. (2022), focusing on the human placenta and developmental toxicity, argue that animal models cannot replicate the unique physiology of human pregnancy. These authors catalogue a range of alternative models (ex vivo placental perfusion, in vitro trophoblast cultures, and computational simulations) and ultimately design a human feto-placental organ-on-chip platform as a next-generation tool for studying prenatal exposures. Their rationale is explicitly linked to ethics (reducing reliance on ethically fraught animal studies involving fetuses or primates) and to scientific yield (gaining mechanistic insight into human-specific developmental processes). In short, Cluster 3 literature illustrates an optimistic picture. By recreating key aspects of human biology in controlled systems, we can obtain more relevant data faster, while sparing animal lives and resources.

However, this cluster also discusses the challenges and requirements for these advanced models to fully deliver on their promise. A recurrent concern is standardization and quality control. Because these systems (organoids, 3D cultures, etc.) are complex and often handcrafted, ensuring reproducibility between labs or batches is difficult. Lee et al. (2025) emphasized the importance of robust quality control (QC) protocols, ranging from genetic stability checks in cell lines to functional assays (e.g., barrier integrity in organoids), to ensure that results are reliable and can be compared across studies. They stress that, without consistent standards, regulatory agencies will remain hesitant to trust data from these models. Regulatory alignment is a persistent subtheme. Many authors note that while in vitro models are improving, formal acceptance in safety testing requires demonstrating their validity and reproducibility on a larger scale. Königer et al. (2024) address one practical barrier, meaning the cost and labor of producing complex 3D tissues, by introducing Robotic Enabled Biological Automation (ReBiA) for tissue culture. By automating cell culturing processes with robotics, ReBiA achieved more consistent and cost-effective production of human tissue models, such as reconstructed skin and airway epithelia, which matched the quality of manually grown cultures. This technological solution directly addresses sustainability and scalability. Automation can lower the expertise threshold and reduce the expense of using in vitro tissues, thereby broadening their adoption and potentially replacing more animal tests. Similarly, authors discuss the need for a robust data infrastructure and data sharing. Carnesecchi et al. (2023) and others argue that harmonizing how NAMs results are reported (through templates and databases) will enable laboratories and regulators to pool knowledge, thereby accelerating the validation and acceptance of new models. In effect, the field recognizes that to integrate these cutting-edge methods into regulatory pipelines, the methods must be as transparent and robust as the traditional assays they aim to replace.

Ethics remains a clear driver throughout Cluster 3, often mentioned together with scientific rationale. It is widely noted that public and regulatory demand for animal-free approaches is especially intense in specific domains such as cosmetics and chemical testing in the EU, or any testing involving higher mammals. Nakamura et al. (2018) reviewed alternative models in skin aging research, pointing out that not only did the EU ban animal testing for cosmetics, but scientifically, animal skin differs markedly from human skin. They described how these pressures led to an array of in vitro skin models (2D cell layers, 3D reconstructed human skin, etc.) that were increasingly indispensable in dermatology research. Yet, they also acknowledged limitations: even popular 3D skin models might lack certain immune components or a fully mature structure. The implication is that continued refinement is needed, possibly integrating in silico simulations or genome-based approaches, to achieve parity or superiority to animal tests eventually (Nakamura et al. 2018). Another ethical facet in this cluster is the consideration of donors and human-derived materials. As we adopt human tissue models, new ethical questions arise regarding the source of cells (e.g., use of embryonic stem cells or gene-edited cells) and the consent and privacy of donors. Neziri et al. (2024) highlight these aspects, noting that organoid use brings concerns like donor consent, potential for human-like sentience in advanced neural organoids, and ownership of biospecimens. Such considerations underscore that the ethics of toxicology now extend beyond animal welfare to include the responsible use of human-derived biology. Nonetheless, the consensus is that these concerns are manageable with proper ethical frameworks and do not diminish the fundamental benefit that human-based NAMs address, namely the moral and scientific problems associated with animal testing.

Sustainability also appears in Cluster 3, often linked to efficiency gains from high-tech models. Efficient cell-based assays can test many more chemicals in a shorter time than animal studies, aligning with the need to assess thousands of legacy chemicals under tight timelines (as mandated by environmental regulations). For instance, Basu et al. (2025) report that regulatory agencies are turning to in vitro and in silico methods to streamline chemical hazard assessment in ecotoxicology, given the high number of substances to evaluate. Basu et al. (2025) define NAMs broadly to include any technology that reduces animal use while improving speed or efficiency of assessments, from cell assays to computational models. This reflects a practical reality: sustainable toxicology is not just about being “green” in an environmental sense, but also about developing scalable testing strategies that can handle modern workloads without relying on slow, costly animal studies. A concrete example is Debad et al. (2025), who documented a recent FDA workshop on developmental neurotoxicity testing. They demonstrated how in vitro neural models and certain non-mammalian species (such as *Caenorhabditis elegans* or zebrafish embryos) were being integrated into testing batteries, vastly increasing throughput for chemical screening and reducing both time and animal usage. Notably, these approaches were discussed as more economical, scalable, and ethical. Thus, the “Culture” cluster demonstrates that technical innovation can serve the triple aim of ethics, scientific advancement, and efficiency.

In summary, Cluster 3 (“Culture”) illustrates the tangible tools of the animal-free testing paradigm and the evolving ecosystem around them. The dense grouping of terms like “cell,” “in vitro,” “human cell,” or “tissue engineering” in our bibliometric map indicates the central role these concepts play in current research. Each term connects to a body of work driving toxicology toward human-mimetic systems, ranging from organ-specific models (such as liver spheroids and beating cardiac tissues) to holistic platforms (including multi-organ “human-on-chip” microphysiological systems). The reviewed literature highlights key breakthroughs, including fully robotic cell culture and functional human organoids for disease modeling. It critically examines what is needed next: increased standardization, more comparative studies to validate these models, more explicit regulatory guidance for their integration, and engagement with emerging ethical issues, including equitable access to technology and the responsible use of human cells.

Ethics and sustainability run as cross-cutting threads. Innovations are framed in terms of how they reduce animal use and improve human relevance, as well as how they can save time, cost, or resources. Taken together, the cluster underscores a broader point: that is, the movement toward animal-free toxicology is not merely an abstract ethical stance, but is being built brick by brick through scientific ingenuity. By “culture,” we refer not only to traditional laboratory cell cultures, but also to the culture of science practice that is increasingly embracing these new methods. As these models become more prevalent, a positive feedback loop is likely to emerge (and is highlighted in our overlay analysis): the more recent publications (dark green to blue in the overlay) tend to cluster around concepts such as “organ-on-chip”, “sustainability”, and “ethical” considerations. This suggests that the latest research is actively integrating technical development with ethical and sustainable frameworks, encompassing the concept of responsible innovation in toxicology.

### Overlay and density visualization interpretation

Overlay and density visualization maps from the bibliometric analysis provide the temporal and thematic context for the evolution of NAMs. High-density areas in the map, marked by terms such as “toxicity”, “in vitro”, and “animal welfare”, confirm the maturity and centrality of these themes across the literature. These represent established zones of inquiry within the “Testing” and “Culture” clusters.

By contrast, overlay maps (which color-code terms by average publication year) reveal emerging keywords, such as “green toxicology”, “responsible innovation”, and “chip”, positioned at the periphery. Their relatively recent appearance in literature points to underexplored frontiers in sustainability, bioengineering, and interdisciplinary ethics. The “Factor” cluster appears to occupy this transitional zone, suggesting that both technical and cultural shifts are actively unfolding.

These visualizations support the interpretation that the NAMs field is expanding from its original ethical and toxicological roots into areas increasingly shaped by sustainability, human systems modeling, and innovation policy. The bibliometric evidence reinforces our cluster-based thematic synthesis.

### Cross-cluster synthesis and matrix of core dimensions

Each cluster of this bibliometric analysis highlights different facets of the transition to ethical, sustainable toxicology, but there are also clear interconnections. Ethical imperatives (animal welfare, the 3Rs, and now the 4th R of responsibility) and the demand for sustainability (reducing resource use and pollution) spread through all three clusters, serving as common motivators. Meanwhile, methodological dimensions such as ensuring human relevance, achieving scientific validity, and securing regulatory acceptance are recurring focal points, even if manifested differently in each cluster. Table 2 provides an overview linking the clusters to several core dimensions that emerge across the literature. Each cell qualitatively indicates the emphasis of that dimension in the cluster, based on our synthesis of the rapid review.

While this review highlights the growing momentum toward ethical and sustainable toxicology, it also reveals important areas where rhetoric and practice have yet to align fully. The prominence of ethical language in the NAMs literature reflects shifting societal ethical values and increasing public and regulatory expectations (Grinnell 2008). However, the practical integration of ethics into methods, validation standards, and implementation frameworks remains uneven.

Ethics is often invoked as a symbolic alignment with funding priorities or public sentiment, rather than as a deeply embedded methodological principle. Nevertheless, emerging work suggests this is beginning to change. For instance, Liu et al. (2025) introduce a fourth R, “responsibility”, reframing ethics as a practical imperative guiding everyday research decisions. Herrmann et al. (2019) call for ethics-informed biomedical design, while Mondou et al. (2020) and Crawford et al. (2017) link ethical reflection to stakeholder engagement and sustainable chemical governance.

Recent initiatives are increasingly operationalizing ethics in tangible ways. Through projects like ONTOX, Vinken et al. (2021) demonstrate how ethical values can shape AI-driven tools for non-animal risk assessment. Similarly, Roy et al. (Roy et al. 2025) outline a “3C” strategy (cell culture, computer simulation, and clinical trials) as an ethical alternative to traditional preclinical testing. These developments indicate that the field is not only articulating an ethical vision, but also beginning to embed it within its scientific and regulatory architecture. Yet, to ensure that this transition is robust and lasting, ethics must be tied to mechanisms of accountability, transparency, and stakeholder participation. Otherwise, there is a risk that ethics will remain a surface-level narrative, rather than a driving force of innovation. The trajectory is encouraging, but realizing truly humane and sustainable toxicology will require efforts to make ethics actionable through reflexive practices, concrete standards, and institutional commitment. This is not a detour from scientific progress, but a necessary condition for its relevance and legitimacy.

### Limitations and future research

While this review provides a comprehensive mapping of ethical and sustainability dimensions in NAMs-related toxicology literature, certain limitations must be acknowledged. First, although WoS and Scopus offer broad interdisciplinary coverage, partially excluding PubMed may have led to the omission of some relevant biomedical research. It is worth emphasizing once again that Scopus covers most of PubMed’s content by indexing MEDLINE (the central and most rigorously curated component of PubMed), thereby ensuring substantial overlap between the two databases.

Next, the present research does not include gray literature. Reliance on keyword-based bibliometric methods risks overlooking thematic nuance in papers that do not explicitly use NAMs-related terminology. Moreover, while bibliometric clusters revealed strong conceptual linkages between ethics, sustainability, and innovation, further empirical studies are needed to assess how these values are being operationalized in practice, particularly in model validation, regulatory decision-making, and stakeholder engagement. Finally, as the field evolves, more attention should be given to emerging ethical concerns such as donor consent in human cell sourcing and equitable global access to advanced NAMs technologies.

## Conclusion

Overall, the discussion across clusters demonstrates that achieving ethical and sustainable transitions in toxicology is a multifaceted endeavor. It requires ethical leadership, scientific innovation, policy reform, and cultural change in tandem. The clusters are distinct, but synergistic pieces of a larger puzzle. Cluster 1 provides a high-level vision and regulatory frameworks that declare why and in what direction we must change. Cluster 2 identifies the critical factors and obstacles that determine how effectively we change. Cluster 3 delivers the concrete scientific means by which change happens. By examining the literature through these lenses, we see that the movement toward NAMs is not only a research trend, but also a reflection of evolving societal values in science. Toxicology, which focuses on the effects of toxic substances on humans and animals, is increasingly expected to safeguard both human health and ecosystems, without relying on animal testing. This growing expectation is driving innovation across the field, from computational modeling to robotic cell culture.

As the present bibliometric overlay suggests (with newer terms such as “sustainability” and “responsible innovation” clustering with traditional toxicology keywords), the community is actively redefining what constitutes “good science” (Bird 2014) in the twenty-first century. It is science that is effective, efficient, ethical, and ecologically conscious. The rapid development of NAMs, documented by thousands of papers and major international initiatives, suggests that this new paradigm is solidifying.

In parallel with its growing role in chemical safety and toxicity testing, NAMs are quickly gaining importance in broader biomedical research areas. Due to ethical concerns, limited translatability of animal models, and the availability of advanced technologies, both regulatory agencies and research communities are rethinking experimental approaches. In space research (Vinken et al. 2025), for instance, human-relevant models such as organoids, bioprinted tissues, and microfluidic organ-on-chip systems are being used to study the physiological effects of microgravity and cosmic radiation, which were traditionally explored using animals. Similarly, the NIH (2025) has launched a strategic initiative to expand human-based technologies and decrease dependence on animal studies, highlighting the importance of models that provide better human relevance and translational accuracy. Tools such as computational simulations, tissue chips, and real-world patient data are now essential parts of the biomedical research toolkit. These changes align with broader European strategies that prioritize 3Rs-aligned innovation and the development of alternatives across various research sectors (European Commission 2025). The broader adoption of NAMs beyond toxicology indicates a systemic shift in the life sciences, reinforcing the ethical, scientific, and societal momentum supporting their development.

Continued interdisciplinary collaboration will be essential to address the remaining gaps (e.g., complex systemic toxicity, long-term exposure effects, and harmonization of global regulations), but the trajectory is clear. The ethical and sustainable transition in toxicology is underway, and each cluster of scholarship contributes to making toxicological science more humane, relevant, and resilient for the future.

## Data Availability

Data used for this review (the collection of references) are available on request from the first or corresponding authors.
